# Feasibility of the Epiduroscopy Simulator as a Training Tool: A Pilot Study

**DOI:** 10.1155/2020/5428170

**Published:** 2020-04-28

**Authors:** Jong Joo Lee, Junho Ko, Yeomin Yun, Seong-Wook Jang, Yoon Ha, Yoon Sang Kim, Dong Ah Shin

**Affiliations:** ^1^Department of Neurosurgery, Bundang Jesaeng Hospital, Seongnam, Republic of Korea; ^2^BioComputing Laboratory, Institute for Bioengineering Application Technology, Department of Computer Science and Engineering, Korea University of Technology and Education (KOREATECH), Cheonan, Republic of Korea; ^3^Department of Neurosurgery, Yonsei University College of Medicine, Seoul, Republic of Korea

## Abstract

Epiduroscopy is a type of spinal intervention that visualizes the epidural space through the sacral hiatus using a fiberoptic scope. However, it is technically difficult to perform compared to conventional interventions and susceptible to complications. Surgery simulator has been shown to be a promising modality for medical education. To develop the epiduroscopy simulator and prove its usefulness for epiduroscopy training, we performed a case-control study including a total of 20 physicians. The participants were classified as the expert group with more than 30 epiduroscopy experiences and the beginner group with less experience. A virtual simulator (EpiduroSIM™, BioComputing Lab, KOREATECH, Cheonan, Republic of Korea) for epiduroscopy was developed by the authors. The performance of the participants was measured by three items: time to reach a virtual target, training score, and number of times the dura and nerve are violated. The training score was better in the expert group (75.00 vs. 67.50; *P* < 0.01). The number of violations was lower in the expert group (3.50 vs. 4.0; *P* < 0.01). The realism of the epidural simulator was evaluated to be acceptable in 40%. Participants improved their simulator skills through repeated attempts. The epiduroscopy simulator helped participants understand the anatomical structure and actual epiduroscopy.

## 1. Introduction

Epiduroscopy is a type of spinal intervention that visualizes the epidural space through the sacral hiatus using a fiberoptic scope [1, 2]. Epiduroscopy has been used to diagnose and treat spinal pathologies by direct visualization and drug delivery [3]. Therapeutic indications include lysis of epidural adhesions, direct application of drugs, and irrigation of inflammatory mediators. It is also useful to place catheter systems and stimulation electrodes and perform tissue biopsies [3]. However, it is technically difficult to perform compared to conventional interventions and susceptible to complications if not properly applied like other endoscopic procedures [4]. It has been reported that epiduroscopy should not be attempted alone by a novice surgeon [5]. Compared to open surgery, epiduroscopy allows limited range of motion of instruments, loss of depth perception, and different surgical views. Nevertheless, the incidence of complications from epiduroscopy has been known to be low when performed by well-trained physicians [4]. However, the training of pain physicians is becoming increasingly difficult because of ethical problems that limit practicing on real patients and work-hour restrictions that reduce training time [6].

Surgery simulator was introduced as early as 1980s by game companies such as Atari [7]. However, it was not until 1990s that surgery simulators became realistic due to graphical development [7]. Simulator training has been shown to be a promising modality for medical education in terms of better task orientation, more accurate metrics, and faster feedback [8]. The advantage of simulator training is that it can avoid the potential risks of practicing on real patients in medical education. It is less cumbersome than cadaver experiments and avoids the ethical problems of animal experiments. Simulator training has been actively used in various fields of medicine, especially in laparoscopic surgery [9, 10]. However, there are a limited number of surgical simulators for spinal interventions [8, 11–15]. To the best of our knowledge, there is no computer simulator for epiduroscopic procedure yet.

Thus, we developed a computer simulator with a three-dimensional (3D) software and joystick interface for epiduroscopy training. The purpose of this study was to develop the epiduroscopy simulator and to prove its usefulness for epiduroscopy training.

## 2. Materials and Methods

### 2.1. Epiduroscopy Simulator

**Figure 1:**
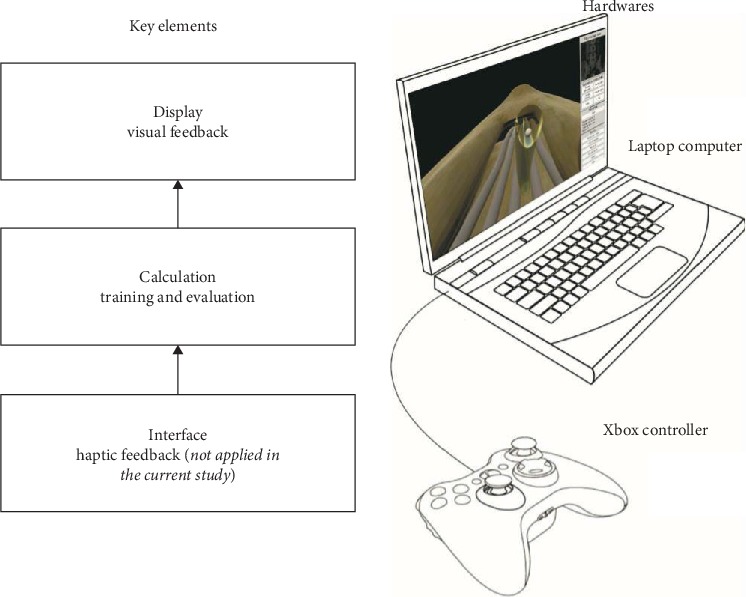
This Schematic illustration of the simulator system. The simulator receives inputs from a joystick, simulates and calculates on a laptop computer, and provides visual feedback to the laptop display.

**Figure 2:**
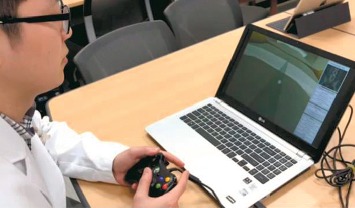
Simulator setup. A physician is practicing epiduroscopy using our simulator system (EpiduroSIM™, BioComputing Lab, KOREATECH, Cheonan, Republic of Korea).

**Figure 3:**
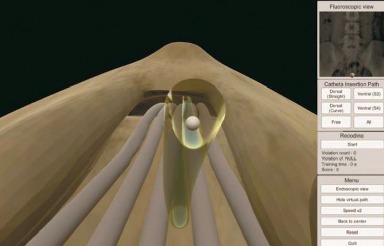
The screen displays four sections: an epiduroscopic view, a fluoroscopic view, a recording section, and a menu bar. The epiduroscopic view is a perspective view that is slightly different from what is seen in real epiduroscopy.

A virtual simulator (EpiduroSIM™, BioComputing Lab, KOREATECH, Cheonan, Republic of Korea) for epiduroscopy was developed to train physicians to perform robotic epiduroscopy that we had developed previously (in press). Robotic epiduroscopy uses a robotic arm to handle epiduroscopy and a manipulating joystick. Before performing robotic epiduroscopy, operator needs simulation training for using the joystick and a computer-generated virtual environment. We thought that this would be also useful for the training of manual epiduroscopy. As a first step, a virtual spine was generated from a DICOM set of human computed tomography data. The DICOM file was converted to 3D geometry by using Mimics software (Materialise Inc., Leuven, Belgium). The 3D geometry was transferred to the 3D rendering software (Unity 3D, Unity Technologies, CA, USA), and the virtual simulator was built on the 3D spine. The epiduroscopy model consists of the spinal canal, intervertebral discs, dural sac, and spinal nerves, which are essential for epiduroscopy training. The epiduroscopy simulator consists of three parts: visualization, training, and evaluation. The hardware of the simulator consists of a laptop and joystick (Figure 1). The simulator uses a joystick to control the epiduroscope during simulation (Figure 2). The display provides both epiduroscopic and fluoroscopic views (Figure 3). The epiduroscopic view provides a view similar to the real epiduroscopic view. The fluoroscopic view provides a view similar to the C-arm view used in real intervention. All parameters are recorded in a file separately in each simulation. The simulator provides the trainee with a training scenario to navigate the catheter to the lesion site. The tasks were designed to develop clinically relevant navigation skills. The assessment items include the time to completion and the number of violations of the nerve and dura and the completion of the mission.

### 2.2. Simulator Practice and Survey

To evaluate the features and usefulness of the simulator, a user experiment was conducted. The ethical approval was achieved from Yonsei University Institutional Review Board (IRB No: 4-2017-0378). Informed consent of the research was obtained from all subjects. A total of 20 physicians participated in the study. The participants were classified as the expert group with more than 30 epiduroscopy experiences and the beginner group with less experience. The following parameters were recorded and analyzed: participant's age, sex, dominant hand, and practice experience. A quick guide on the experiment was given to all participants. The participants were allowed to handle the system freely for 5 min to get familiar with it. Then, the participants participated in the final evaluation test. Each participant performed the simulator 20 times in the same day. The rules of the simulation test were as follows: The field of view was the view seen with epiduroscopy. It was assumed that the sacral hiatus had been punctured with a Tuohy needle, and the epiduroscope had already been placed in the epidural space. The tip of the epiduroscopy should start at the sacral hiatus and reach the posterior part of the L4–5 intervertebral disc through the epidural space. The purpose of the mission was to place the tip of the catheter on the target point as fast as possible without touching the nerve and the dura. The training score of EpiduroSIM™ was scored from the initial score of 100 points. During the experiment, 5 points were deducted for a collision between the catheter and the dura or nerve and 5 points were deducted every 30 seconds from the game start. If the time limit of 5 min exceeded, a final score of 30 points was deducted. After simulation test, the participants were requested to complete the questionnaire. The questionnaire was modified based on a previous study [15]. It was composed of three sections. The participants were asked to grade the items on an ordinal scale of 5, with 5 being the strongest agreement and 1 being the strongest disagreement. The items and results are summarized in Table 1.

### 2.3. Statistical Analysis

The chi-square test was used for nominal variables, and the Mann–Whitney test was used for continuous variables to compare between beginner and expert groups by epiduroscopy experience. All statistical analyses were performed using SPSS software version (SPSS Inc., Chicago, IL, USA) and statistical significance was defined as *P* < 0.05.

## 3. Results

The study group was composed of 20 physicians with a mean age of 41.00 years (Table 2). They were all neurosurgeons, and they were both performing surgical and nonsurgical treatments for spinal pain. Seven physicians were included in the beginner group, and 13 physicians were included in the expert group. As shown in Table 3, the expert group was older than the beginner group (44.00 vs. 35.00 years; *P* < 0.01). All participants were men and right-handed. The practice experience was longer in the expert group (*P* < 0.01).

**Figure 4:**
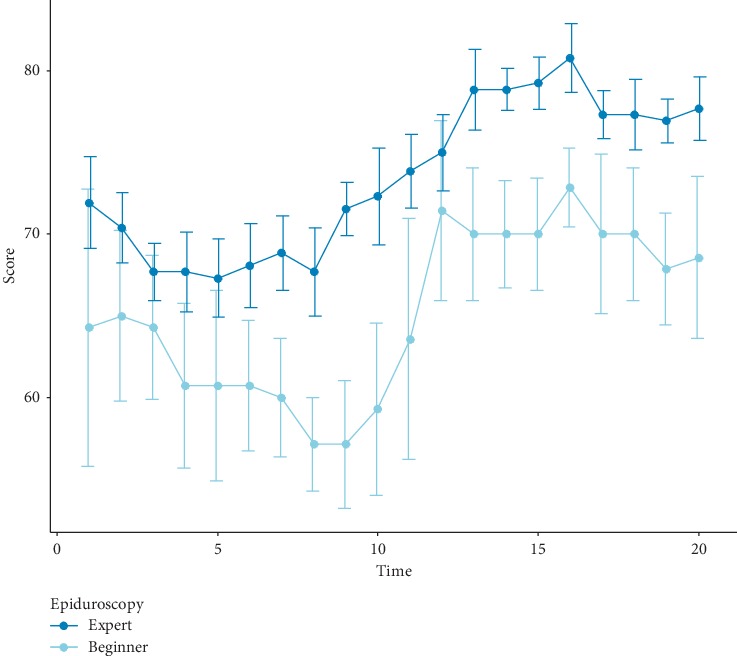
Comparison of the total score between experts and beginners. The average score of the expert group was higher than that of the beginner group during the whole experiment period, indicating that the simulator can distinguish different levels of experience. The score of the beginner group was significantly improved after 10 experiments.

The experimental results are shown in Table 4. The performance of the participants was measured by three items: time to reach a virtual target, training score, and the number of violations of the dura and nerve. As shown in Figure 4, the simulator can distinguish between different levels of experience. While there was no difference in the time to reach a virtual target between both groups with a median time of 83.50 s vs. 83.00 s, the training score was better in the expert group (67.50 vs. 75.00; *P* < 0.01), and the number of violations was lower in the expert group (4.00 vs. 3.50; *P* < 0.01).

The survey results are summarized in Table 1. The degree of the similarity to the real anatomy and degree of the similarity with the actual epiduroscopy were evaluated to be acceptable in 40%. These could be improved in the future studies. However, 75% agreed that the simulator training was helpful in anatomical understanding and 85% agreed that the simulator was helpful in epiduroscopy training. As documented, the participants requested better visual realism and additional haptic function.

## 4. Discussion

Our simulator was useful in teaching the epiduroscopy procedure to physicians. It allowed physicians to practice epiduroscopy in a virtual environment. It converted a physician's performance of virtual epiduroscopy into a score. The simulator improved the performance of the physicians after repeated practice, and they reported the usefulness of the virtual simulator.

Various simulators have been used for physician training, noninvasive assessment, and preoperative planning, as well as for real practice [15–17]. Accessibility, repeatability, and safety, which are characteristics of simulator training, make this an ideal educational tool. [16]. Simulator training has tremendous potential for improving interventional skill. However, simulator studies in the field of spinal intervention are still limited [8, 11, 12, 16]. To the best of our knowledge, our system is the first virtual simulator of spinal epiduroscopy.

Previous simulators were commonly running on mock-ups similar to real anatomy [9, 12, 15, 18, 19]. However, we implemented epiduroscopy simulation graphically in a computer. Real epiduroscopy is performed while viewing images of the epidural space on a two-dimensional (2D) monitor. Thus, we believe that simulation exercise using our simulator with the 2D monitor will enhance understanding of the real epiduroscopic view. In addition, our simulator uses a joystick for a control unit. It will help to enhance spatial attention and eye–hand coordination like that in video games [10, 20].

However, additional equipment is required to operate the simulator, which can be considered a disadvantage. Some simulators require additional space to be installed and are expensive. Furthermore, it is not available for commercial purchase. Nevertheless, the development of the epiduroscopy simulator is essential because epiduroscopy has a steep learning curve. In addition, practicing on a real patient not only creates ethical problems, but also is not a low–cost training method when considering possible complications. Unavailability, ethical concerns, higher costs, time consumption, and potential hazards of cadavers are some of the aspects that lead to their limited usage in surgical course [16, 21]. Therefore, it would be of great advantage to be able to practice indefinitely without the restriction of time or place [16].

This study has several limitations. First, some participants evaluated that they were not satisfied with the anatomical similarity of our simulator. They criticized that the anatomical details were lacking and the point of view was unnatural. This will be revised and supplemented in the next study by improving the graphical interface. Second, a haptic device was developed in this study, but it was not used because of a technical limitation in actual experiments. The haptic device will be installed in the simulator in the future study. Third, this study included a small number of participants; therefore, we plan to conduct a large–scale study by recruiting a large number of subjects in the future.

## 5. Conclusions

In this study, the authors developed a virtual simulator for epiduroscopy training. Participants improved their simulator skills through repeated practice. The epiduroscopy simulator helped participants understand the anatomical structure and the actual epiduroscopy procedure. Future research will be required to demonstrate that simulator practice leads to improved performance of actual epiduroscopy.

## Figures and Tables

**Table 1:** Survey responses on the features and usefulness of the simulator.

	Beginner group	Expert group	Total
*The 3D virtual environment is similar to the real epidural anatomy*			
Strongly disagree	1 (14.3%)	0 (0.0%)	1 (5.0%)
Disagree	0 (0.0%)	2 (15.4%)	2 (10.0%)
Neutral	3 (42.9%)	6 (46.2%)	9 (45.0%)
Agree	2 (28.6%)	4 (30.8%)	6 (30.0%)
Strongly agree	1 (14.3%)	1 (7.7%)	2 (10.0%)
*The simulator is similar to the real epiduroscopy*			
Strongly disagree	2 (28.6%)	2 (15.4%)	4 (20.0%)
Disagree	2 (28.6%)	1 (7.7%)	3 (15.0%)
Neutral	0 (0.0%)	5 (38.5%)	5 (25.0%)
Agree	2 (28.6%)	4 (30.8%)	6 (30.0%)
Strongly agree	1 (14.3%)	1 (7.7%)	2 (10.0%)
*The training is helpful in anatomical understanding of the catheter insertion pathway (spatial awareness)*			
Strongly disagree	1 (14.29%)	0 (0%)	1 (5.0%)
Disagree	0 (0.0%)	1 (7.7%)	1 (5.0%)
Neutral	1 (14.3%)	2 (15.4%)	3 (15.0%)
Agree	2 (28.6%)	8 (61.5%)	10 (50.0%)
Strongly agree	3 (42.9%)	2 (15.4%)	5 (25.0%)
*The simulator training will be helpful in the training or education of real epiduroscopy*			
Strongly disagree	1 (14.3%)	0 (0.0%)	1 (5.0%)
Disagree	0 (0.0%)	0 (0.0%)	0 (0.0%)
Neutral	0 (0.0%)	2 (15.4%)	2 (10.0%)
Agree	2 (28.6%)	6 (46.2%)	8 (40.0%)
Strongly agree	4 (57.1%)	5 (38.5%)	9 (45.0%)

Both groups responded favorably to the functionality of the simulator system.

**Table 2:** Features of the presented simulator (EpiduroSIMTM, BioComputing Lab, KOREATECH, Cheonan, Republic of Korea).

Items	Features
Operating system	Windows XP (Microsoft, CA, USA)
Software	Unity 3D (Unity Technologies, CA, USA)
Hardware	Laptop computer (i3 CPU, 2 gb RAM, 500 gb HDD)
Display	RGB, 32 bit color depth
Resolution	1024 × 768
Control interface	Xbox controller (Microsoft, CA, USA)
Haptic training	Applicable, but not applied in this study
Obtainable data	Time to appropriate position of catheter, total score, and number of violations

**Table 3:** Participant characteristics.

	All participants (*n* = 20)	Beginner group (*n* = 7)	Expert group (*n* = 13)	*P* value
Mean age (years) (median, IQR)	41.00 (12)	35.00 (11)	44.00 (10)	<0.01
Sex, male (%)	20 (100)	7 (100)	13 (100)	—
Dominant hand, right-handed (%)	20 (100)	7 (100)	13 (100)	—
*Clinical experience regardless of epiduroscopy (%)*				
0–2 years	7 (35.0)	2 (28.6)	5 (38.5)	<0.01
3–5 years	4 (20.0)	0 (0)	4 (30.8)	
5–9 years	6 (30.0)	4 (57.1)	2 (15.4)	
10 years	3 (15.0)	1 (14.3)	2 (15.4)	

Mann–Whitney test, chi-square test.

**Table 4:** Comparison of the simulation performance between the beginner and expert groups.

	All participants (*n* = 20)	Beginner group (*n* = 7)	Expert group (*n* = 13)	*P* value
Time to reach a virtual target (median, IQR) (s)	83.00 (10)	83.50 (10)	83.00 (10)	0.194
Training score number of violations (median, IQR)	70.00 (20)	67.50 (15)	75.00 (15)	<0.01
Dura (*n*)	3 (2)	4.00 (2)	2.50 (1)	<0.01
Nerve (*n*)	0 (1)	0.00 (1)	1.00 (1)	<0.01

Mann–Whitney test.

## Data Availability

The data used to support the findings of this study are available from the corresponding author upon request.
